# Ensembl 2017

**DOI:** 10.1093/nar/gkw1104

**Published:** 2016-11-29

**Authors:** Bronwen L. Aken, Premanand Achuthan, Wasiu Akanni, M. Ridwan Amode, Friederike Bernsdorff, Jyothish Bhai, Konstantinos Billis, Denise Carvalho-Silva, Carla Cummins, Peter Clapham, Laurent Gil, Carlos García Girón, Leo Gordon, Thibaut Hourlier, Sarah E. Hunt, Sophie H. Janacek, Thomas Juettemann, Stephen Keenan, Matthew R. Laird, Ilias Lavidas, Thomas Maurel, William McLaren, Benjamin Moore, Daniel N. Murphy, Rishi Nag, Victoria Newman, Michael Nuhn, Chuang Kee Ong, Anne Parker, Mateus Patricio, Harpreet Singh Riat, Daniel Sheppard, Helen Sparrow, Kieron Taylor, Anja Thormann, Alessandro Vullo, Brandon Walts, Steven P. Wilder, Amonida Zadissa, Myrto Kostadima, Fergal J. Martin, Matthieu Muffato, Emily Perry, Magali Ruffier, Daniel M. Staines, Stephen J. Trevanion, Fiona Cunningham, Andrew Yates, Daniel R. Zerbino, Paul Flicek

**Affiliations:** 1 European Molecular Biology Laboratory, European Bioinformatics Institute, Wellcome Genome Campus, Hinxton, Cambridge CB10 1SD, UK; 2 Wellcome Trust Sanger Institute, Wellcome Genome Campus, Hinxton, Cambridge, CB10 1SA, UK

## Abstract

Ensembl (www.ensembl.org) is a database and genome browser for enabling research on vertebrate genomes. We import, analyse, curate and integrate a diverse collection of large-scale reference data to create a more comprehensive view of genome biology than would be possible from any individual dataset. Our extensive data resources include evidence-based gene and regulatory region annotation, genome variation and gene trees. An accompanying suite of tools, infrastructure and programmatic access methods ensure uniform data analysis and distribution for all supported species. Together, these provide a comprehensive solution for large-scale and targeted genomics applications alike. Among many other developments over the past year, we have improved our resources for gene regulation and comparative genomics, and added CRISPR/Cas9 target sites. We released new browser functionality and tools, including improved filtering and prioritization of genome variation, Manhattan plot visualization for linkage disequilibrium and eQTL data, and an ontology search for phenotypes, traits and disease. We have also enhanced data discovery and access with a track hub registry and a selection of new REST end points. All Ensembl data are freely released to the scientific community and our source code is available via the open source Apache 2.0 license.

## INTRODUCTION

Over the past several years, large-scale genomics consortia have come together to address key biological questions by creating datasets of sufficient size and scope that they become widely used references. These efforts include the 1000 Genomes Project (1), ENCODE (2), the Gene-Tissue Expression (GTEx) project (3), the Exome Aggregation Consortium (ExAC) (4), the Mouse Genomes Project (5) and the various component projects of the International Human Epigenome Consortium (IHEC). The data and results from these projects have created a strong foundation on which genomics research can build.

The Ensembl project was originally founded to annotate the human genome and has grown into a central hub of genomic information. When a new genome assembly is included in Ensembl, we integrate diverse data to produce a collection of Ensembl resources for gene annotation (6), genome variation (7), gene regulation (8) and comparative genomics (9).

We also develop and distribute a suite of databases, tools (10,11), APIs (12,13) and web interfaces (14) for generating, querying and distributing these data and in doing so we ensure consistent data analysis and access for all of our species.

The outputs of the large-scale projects listed above are important components within the overall collection of Ensembl resources. By integrating all of these genomics data resources into a coherent informatics infrastructure we enable further research by simplifying and standardizing the methods for data access and visualization. We also help make these data resources easily accessible to a wide variety of researchers.

Our data and software are updated at regular intervals following a formal release process that ensures data and software provenance tracking via an Ensembl release number. Ensembl release data are archived and can be reliably retrieved into the future. In addition, the release process ensures that data are synchronized across all of Ensembl. For example, updates to the human gene set will trigger updates to the orthologs for all species.

We collaborate with other informatics resources and tools including the Genome Reference Consortium (GRC) (15), the UCSC Genome Browser (16), UniProt (17), model organism databases (18,19) and relevant resources at the NCBI (20) to coordinate data presentation and standards.

We use and support ontological and other standard formats for our data and have worked directly with the Sequence Ontology (SO) to address gaps in the current representations (21). Increasingly, these efforts are taking place in the context of the Global Alliance for Genomics and Health (GA4GH), which works to create interoperable approaches to facilitate genomic data sharing (22). For example, in the past year, we have developed GA4GH-compliant services that offer Ensembl data. These new Ensembl REST endpoints return sequence features, genotype calls, variant annotation, lists of reference sequences and associated metadata in standard GA4GH formats.

In this report we highlight new data and tools for human genome interpretation, with an emphasis on new resources for gene regulation and population genomics. We describe new and updated data for other species, and the accompanying tools and methods for searching, browsing, downloading and analyzing these new features.

## ANNOTATING THE HUMAN GENOME

### Transcriptional regulation

This year, we significantly expanded our catalog of human cell types with evidence-based annotated regulatory elements, which are now available for 68 cell types and tissues as of Ensembl release 86 (October 2016). The increase is largely based on datasets from the IHEC member projects BLUEPRINT (23) and Roadmap Epigenomics (24), which were uniformly annotated using the Ensembl Regulatory Build methodology (25). This process results in a defined location and predicted function for each regulatory element and, for each available cell type or tissue, an activity status such as ‘active’, ‘poised’, ‘repressed’ or ‘inactive’. As a result, we now cover a considerable fraction of the epigenomes thus far generated by ENCODE and IHEC, and we will increase our regulatory annotations as more data become available.

**Figure 1.**
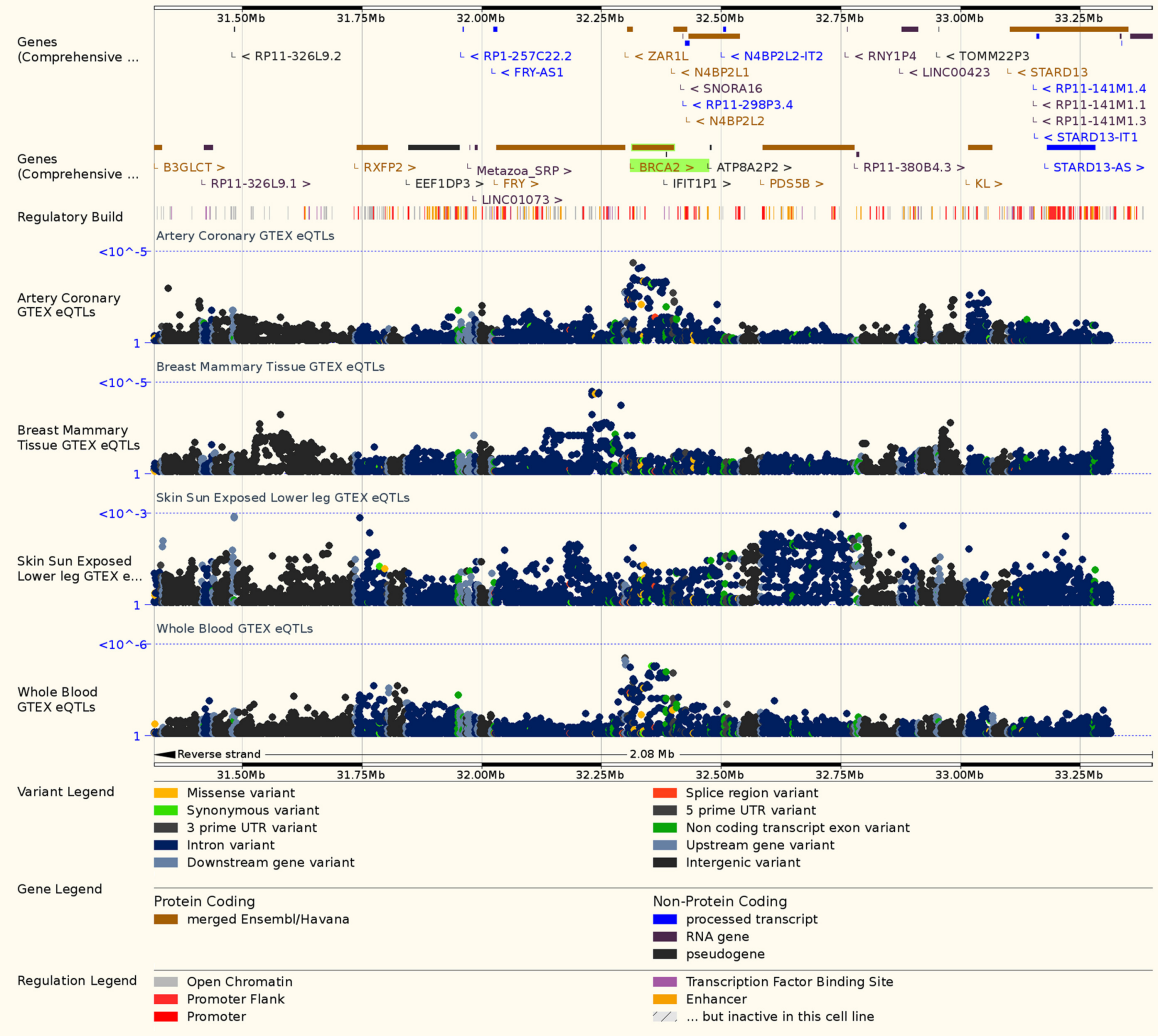
Regulation view. For each human gene, Regulation view displays correlation statistics (eQTLs) between genetic variation and tissue-specific expression. For *BRCA2*, data from the GTEx Project are available as Manhattan plots for over 40 tissues, including the four tissues displayed: Artery Coronary, Breast Mammary Tissue, Skin Sun Exposed Lower Leg, and Whole Blood.

We have also recently incorporated expression quantitative trait loci (eQTL) data from GTEx to provide unfiltered SNP-to-gene correlation statistics from 44 tissues (3). This rich dataset can be viewed on our website (Figure 1) and accessed through our REST API, facilitating advanced post-Genome Wide Association Studies (GWAS) functional analysis without the overhead of handling the associated large data files.

### Gene annotation and transcript haplotypes

Ensembl's primary gene annotation on the latest human reference assembly, GRCh38, is GENCODE. It was updated regularly over the past year, to include manually annotated transcripts and new gene models on the alternate sequence regions defined by the GRC (26). GENCODE remains the most comprehensive human gene set (27–29) and this year's updates have also included our presentation of supporting analyses including APPRIS (30), Transcript Support Levels, and the GENCODE Basic set which can be used to identify a subset of the GENCODE transcripts suitable for most applications.

For each GENCODE transcript, we have also calculated the list of observed haplotypes in the 1000 Genomes Project phase 3 data and present these as a series of alterations from the reference sequence for the transcript's coding sequence and protein product. We also provide haplotype frequencies, by population, for each transcript via our new Transcript Haplotype view. To enable further analysis, alignments of the individual haplotypes against the reference assembly are available and the entire set of sequences and metadata can be downloaded in JSON format.

### Discovery, prioritization and annotation of sequence variants

We now identify small-scale variants (such as insertions and deletions) as ‘equivalent’ on our Variant page when they lead to the same alteration to the reference assembly. Equivalent variants can receive separate accession numbers and nominal genomic mappings in databases such as dbSNP when they occur within lower complexity sequence such as dinucleotide repeats. Identifying these variants is particularly useful when one includes frequency information that could also apply to other nearby variants. For example, rs397714540 had no associated frequency data whereas the equivalent variant rs36021200 does have such data from the 1000 Genomes Project.

To aid prioritizing of variants within a gene, enhanced filtering and sorting is now available for the variant tables on our web site. The new tables can manage many hundreds of thousands of rows, and can be customized to display only variants with a range of SIFT (31) or PolyPhen (32) scores; those with particular consequence types or minor allele frequencies; or other properties.

To facilitate data discovery and querying across our various input sources for phenotype, trait and disease annotations—including ClinVar (33), OMIM Morbid (34), the GWAS Catalog (35) and Orphanet (36)—we now map their descriptions onto the Experimental Factor Ontology (EFO) (37), Human Phenotype Ontology (HPO) (38) and Orphanet Rare Disease Ontology. This process helps to rationalize the different descriptions these resources use for similar concepts. By bringing these together, it is now possible to search Ensembl for a disease or phenotype, and to discover variants associated with its synonyms. For example, a search for ‘Keratosis follicularis’ will now reveal variant rs121912732, which is reported by ClinVar as pathogenic and associated to Darier disease.

## COMPARATIVE GENOMICS AND NON-HUMAN SPECIES

### Confidence scores and visualization options for homology relationships

We added two new confidence scores to the homology predictions that arise from our TreeFam phylogenetic gene trees (39), which are the basis for inferring homology relationships, including within-species and cross-species events such as gene duplication and gene loss. The first confidence score is based on coverage across all genome sequence alignments, including both pairwise and multiple sequence alignments. This score relies on the assumption that high-quality ‘true’ orthologs should be well aligned to each other, and it weights alignments over exons more highly than alignments over introns. The second confidence score is based on how well the local (upstream and downstream) gene order is conserved. This score is based on the observation that evolutionary genome rearrangements are likely to happen to a group of contiguous genes, thereby conserving the local gene order surrounding any one gene. Both scores are displayed in the Orthologues table available from each Gene view page. Together, they make it easier to identify high-confidence orthologs by using them alongside the existing filters, such as a threshold on the percentage of sequence identity.

To explore the protein sequence alignments supporting our gene trees, the GeneTree view (also available from each Gene view page) now provides a link to the Wasabi interactive alignment visualization tool (40).

### Protein family classification

To quickly and accurately infer the function of genes in newly sequenced genomes, we have created a new Hidden Markov Model (HMM) library for matching new protein sequences to existing, well-studied proteins from other species. This HMM library uses the PANTHER families as a base, is supplemented with our own data, and has been defined across all eukaryote genomes, including non-vertebrates in Ensembl Genomes. This HMM library is available for download (ftp://ftp.ensembl.org/pub/current_compara), and provides a stable and scalable means to classify new protein sequences into our protein families resource.

### Mouse strain genomes

**Figure 2.**
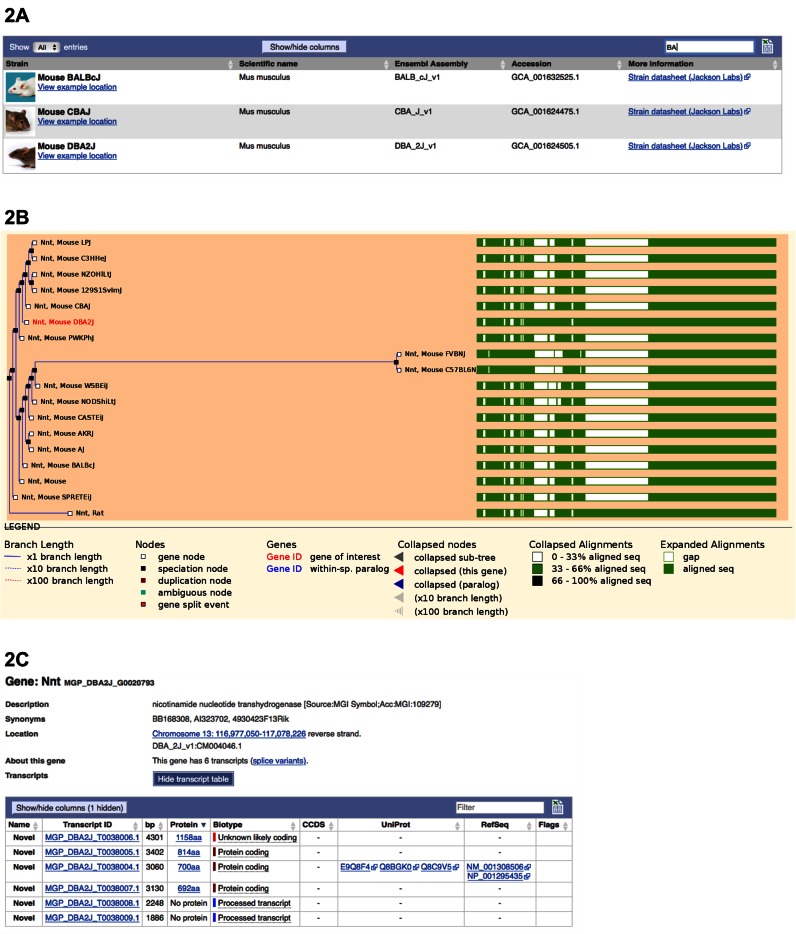
Mouse strains in Ensembl. (**A**). The Mouse Strain landing page (http://www.ensembl.org/Mus_musculus/Info/Strains) lists available assemblies in Ensembl, and includes links to more information about each strain. Mouse strains BALB/cJ, CBA/J, and DBA/2J are among those now available. (**B**) GeneTree view showing sequence conservation for *Nnt* between the mouse strains and the reference mouse and rat. The alignment shows extra sequence in the rat and DBA/2J genes that are not represented in the other mouse annotation. Each gene can be clicked for more information and to navigate to the Gene view. (**C**). Gene view for *Nnt* in mouse DBA/2J. The longest annotated protein is 1158 amino acids in length, whereas the longest annotated protein in the reference mouse (GRCm38) is only 835 amino acids in length.

Whole genome sequencing of key laboratory mouse strains has been ongoing over the last several years (5,41). Following a transition of the Mouse Genomes Project from a resequencing to *de novo* assembly strategy for a core set of 16 inbred mouse strains and subspecies, these mouse assemblies now fit Ensembl's data model and were introduced in Ensembl release 86 (October 2016) (Figure 2). We have annotated repeats, CpG islands, and promoter regions on these assemblies. Gene annotation for the 16 strain assemblies is provided directly by the Mouse Genomes Project using a process of whole genome alignments, annotation projection and various filters. We aligned UniProt proteins and annotated protein features on the protein coding transcripts. We also computed rodent-specific phylogenetic trees (‘gene trees’) on the protein coding genes, and inferred orthologs and paralogs from them.

In contrast to the annotation for the 16 mouse strains, the gene annotation for the C57BL/6J reference mouse genome assembly, GRCm38, is produced by GENCODE. The mouse GENCODE annotation has been updated several times this year and combines the standard Ensembl gene annotation approach (6) with manual annotation directly on the reference assembly.

### Updated chicken genome assembly and annotation

Our chicken resources were updated to the latest chicken assembly, Gallus_gallus-5.0 (GCA_000002315.3), in Ensembl release 86 (October 2016). In a first for any species in Ensembl, we incorporated PacBio Iso-Seq data from brain and embryo libraries to support annotation of alternate splicing. These data supplemented the standard collection of evidence used for annotation including, in this case, protein sequences, cDNA sequences, and Illumina RNA-seq data from 20 different tissues. As with all cases when we update a species to a new assembly, we propagated gene stable identifiers from the old assembly to ensure consistency across the assembly update. All comparative genomics resources for chicken were also updated including the relevant TreeFam gene trees and homology (ortholog and paralog) annotation based on the updated gene annotation and our pairwise whole genome alignments from chicken to 12 other species, including seven birds. Our sauropsid Enredo Pecan Ortheus (EPO) alignments (42,43), and our amniote Mercator Pecan multiple alignments (42,44) were fully recomputed to include the new chicken assembly.

### Annotation for other species

The zebrafish and rat gene sets have both been updated to include manual annotation from HAVANA (45). We annotated additional gene models for zebrafish based on RNA-seq data taken from the embryo at six hours post-fertilization and 24 hours post-fertilization.

Annotation for rhesus macaque and mouse lemur was updated to include the latest assemblies, Mmul_8.0.1 (GCA_000772875.3) and Mmur_2.0 (GCA_000165445.2), respectively. For both primates, we annotated gene models using an improved version of our gene annotation system that produces more transcript variants per gene than the previous version. We also updated the TreeFam gene trees, homology annotation, and pairwise whole genome alignments to human as well as our primates and mammals EPO multiple alignments to include both new primate assemblies.

Finally, we added long intergenic noncoding RNA (lncRNA) genes for seven additional species: dog (*Canis familiaris*), armadillo (*Dasypus novemcinctus*), ferret (*Mustela putorius furo*), anole lizard (*Anolis carolensis*), cave fish (*Astyanax mexicanus*), flycatcher (*Ficedula albicollis*) and olive baboon (*Papio anubis*).

## TOOLS AND INFORMATION FOR GENOME ANALYSIS AND INTERPRETATION

### Variant Effect Predictor

The Ensembl Variant Effect Predictor (VEP) is a tool for annotating and prioritizing genomic variants, and relies on our comprehensive and up-to-date data (46). Significant improvements this year include speed and memory optimizations. We have also implemented powerful new filtering options for the VEP results, including support for nested filters. For example, the following filtering statement is now possible:

GMAF < 0.1 and ((Consequence is missense_variant and (SIFT is deleterious or PolyPhen is probably_damaging)) or Consequence match stop)

To better support RefSeq transcripts (47), VEP now reports information on matched regions between Ensembl and RefSeq transcripts and mismatches between RefSeq transcripts and the reference genome assembly (46).

This year has seen us release new and updated plugins for the VEP, and we continue to encourage the community to submit their VEP plugins to our dedicated GitHub repository (https://github.com/Ensembl/VEP_plugins). To further promote the re-use of these plugins, we have added functionality so that VEP plugins can be run via our website or using our REST API. The offline script version has also been updated to support output of conservation scores and ExAC frequency data (4).

### Population genomics

We have improved the access methods for linkage disequilibrium (LD) data by developing a faster and more robust RESTful API and Perl API method to retrieve LD values between a specific pair of SNPs. We use this method ourselves to display LD values as a Manhattan plot accessible from the Variant pages.

We have also migrated three tools to support genome variation analysis that were previously only available on the 1000 Genomes Brower (48). The Allele Frequency Calculator determines population-wide allele frequencies for sites within the chromosomal region defined from a VCF file and populations defined in a sample file. The VCF to PED Converter transforms a VCF file to a linkage pedigree (PED) file and a marker information file, which together may be loaded into linkage disequilibrium display tools such as Haploview (49). The Variant Pattern Finder identifies shared variation between individuals in a chromosomal region of interest. These tools use data from the 1000 Genomes Project phase 1 and 3 studies, and are currently only available on our GRCh37 archive site. All tools can be accessed via the Tools link at the top of each Ensembl page.

### CRISPR/Cas9 target regions

The CRISPR/Cas9 system has recently inspired a new array of laboratory techniques for targeted genome editing, knock-out screens and functional assays. Short single guide RNA molecules (sgRNA) are used to lead the enzyme to precise genomic locations. However, like PCR primers, not all regions of the genome are as readily accessible and sgRNA sequences with few off-targets sites are more likely to be specific in their binding. To assist experimental design, we annotated the human and mouse genomes with all possible CRISPR/Cas9 single guide RNA binding sites in a new ‘WGE CRISPR sites’ track on our browser's Location view (Region in Detail). Each site can be clicked separately to reveal an information window with specificity statistics produced by the Wellcome Trust Sanger Institute Genome Editing group (50).

### Track Hub Registry

Ensembl has supported display of external datasets stored in track data hubs since 2013 (51) and watched them develop into a popular method for many projects to organize, share and display genome-wide datasets (52). Widespread use of track hubs has made finding relevant data increasingly difficult. To address this, we have designed the Track Hub Registry (http://www.trackhubregistry.org) to catalog and search publicly accessible track hubs. Hubs can be searched and attached via the Track Hub Registry website or from a specialized search from our custom data interfaces.

### File Chameleon

We have developed the File Chameleon tool to help address the perennial bioinformatics problem of ensuring that input files match the format specified by a specific software package. For example, some analysis software requires the ‘chr’ string at the start of a chromosome name, or will not allow genes longer than 2Mb. Pre-processing the input files is time-consuming, requires domain knowledge and could lead to errors. File Chameleon makes downloading customized versions of the files on our FTP site easy. Instead of searching our FTP site, the dataset and format requirements are provided to File Chameleon, which will then produce the correctly formatted files for download. Access to the online version of File Chameleon is at http://www.ensembl.org/Homo_sapiens/Tools/FileChameleon; it is also available as a standalone script (https://github.com/FAANG/faang-format-transcriber) so it can be run locally on any file.

## TRAINING, OUTREACH AND USER SUPPORT

We offer extensive in-person training (http://training.ensembl.org) as well as online courses, live webinars, YouTube tutorials (https://www.youtube.com/user/EnsemblHelpdesk) and static text-based courses. This year saw the first iteration of our live online course (http://www.ebi.ac.uk/training/online/course/ensembl-browser-webinar-series-2016/), consisting of a series of seven live webinars on using the Ensembl website, with accompanying exercises and catch-up videos on the EBI's Train Online platform.

Queries about hosting Ensembl workshops and any other questions about Ensembl can be directed to our helpdesk (helpdesk@ensembl.org). We can also be contacted informally via social media platforms, including Twitter (@ensembl) and Facebook (Ensembl.org). Our blog posts include detailed descriptions of every Ensembl release and other information (http://www.ensembl.info).

## CONCLUSION

Ensembl is a central hub of genomic data that creates and presents high-quality reference datasets in a consistent, accessible infrastructure. Among other updates, over the past year we have expanded our human genome resource with extensive regulatory data and major external datasets and included 16 new mouse strain assemblies. In response to increasing data size and complexity, we expanded our tools and methods for searching, filtering and prioritizing data. New and updated genomes, annotation, datasets and tools are part of every Ensembl release. We believe these efforts will ensure that Ensembl remains a valuable source of data and tools for interpreting biology on assembled genome sequences.

## References

[1] The 1000 Genomes Project Consortium (2015). A global reference for human genetic variation. Nature.

[2] ENCODE Project Consortium (2012). An integrated encyclopedia of DNA elements in the human genome. Nature.

[3] GTEx Consortium (2015). Human genomics. The Genotype-Tissue Expression (GTEx) pilot analysis: multitissue gene regulation in humans. Science.

[4] Lek M., Karczewski K.J., Minikel E.V., Samocha K.E., Banks E., Fennell T., O'Donnell-Luria A.H., Ware J.S., Hill A.J., Cummings B.B. (2016). Analysis of protein-coding genetic variation in 60, 706 humans. Nature.

[5] Adams D.J., Doran A.G., Lilue J., Keane T.M. (2015). The Mouse Genomes Project: a repository of inbred laboratory mouse strain genomes. Mamm. Genome.

[6] Aken B.L., Ayling S., Barrell D., Clarke L., Curwen V., Fairley S., Fernandez Banet J., Billis K., García Girón C., Hourlier T. (2016). The Ensembl gene annotation system. Database (Oxford).

[7] Chen Y., Cunningham F., Rios D., McLaren W.M., Smith J., Pritchard B., Spudich G.M., Brent S., Kulesha E., Marin-Garcia P. (2010). Ensembl Variation Resources. BMC Genomics.

[8] Zerbino D.R., Johnson N., Juetteman T., Sheppard D., Wilder S.P., Lavidas I., Nuhn M., Perry E., Raffaillac-Desfosses Q., Sobral D. (2016). Ensembl regulation resources. Database (Oxford).

[9] Herrero J., Muffato M., Beal K., Fitzgerald S., Gordon L., Pignatelli M., Vilella A.J., Searle S.M.J., Amode R., Brent S. (2016). Ensembl comparative genomics resources. Database (Oxford).

[10] Severin J., Beal K., Vilella A.J., Fitzgerald S., Schuster M., Gordon L., Ureta-Vidal A., Flicek P., Herrero J. (2010). eHive: An Artificial Intelligence workflow system for genomic analysis. BMC Bioinformatics.

[11] Kinsella R.J., Kähäri A., Haider S., Zamora J., Proctor G., Spudich G., Almeida-King J., Staines D., Derwent P., Kerhornou A. (2011). Ensembl BioMarts: a hub for data retrieval across taxonomic space. Database (Oxford).

[12] Stabenau A., McVicker G., Melsopp C., Proctor G., Clamp M., Birney E. (2004). The Ensembl core software libraries. Genome Res..

[13] Yates A., Beal K., Keenan S., McLaren W., Pignatelli M., Ritchie G.R.S., Ruffier M., Taylor K., Vullo A., Flicek P. (2015). The Ensembl REST API: Ensembl Data for Any Language. Bioinformatics.

[14] Parker A., Bragin E., Brent S., Pritchard B., Smith J.A., Trevanion S. (2010). Using caching and optimization techniques to improve performance of the Ensembl website. BMC Bioinformatics.

[15] Church D.M., Schneider V.A., Graves T., Auger K., Cunningham F., Bouk N., Chen H.-C., Agarwala R., McLaren W.M., Ritchie G.R.S. (2011). Modernizing reference genome assemblies. PLoS Biol..

[16] Speir M.L., Zweig A.S., Rosenbloom K.R., Raney B.J., Paten B., Nejad P., Lee B.T., Learned K., Karolchik D., Hinrichs A.S. (2016). The UCSC Genome Browser database: 2016 update. Nucleic Acids Res..

[17] UniProt Consortium (2015). UniProt: a hub for protein information. Nucleic Acids Res..

[18] Howe D.G., Bradford Y.M., Conlin T., Eagle A.E., Fashena D., Frazer K., Knight J., Mani P., Martin R., Moxon S.A. (2013). ZFIN, the Zebrafish Model Organism Database: increased support for mutants and transgenics. Nucleic Acids Res..

[19] Shimoyama M., De Pons J., Hayman G.T., Laulederkind S.J., Liu W., Nigam R., Petri V., Smith J.R., Tutaj M., Wang S.J. (2015). The Rat Genome Database 2015: genomic, phenotypic and environmental variations and disease. Nucleic Acids Res..

[20] NCBI, Resource Coordinators (2016). Database resources of the national center for biotechnology information. Nucleic Acids Res..

[21] Cunningham F., Moore B., Ruiz-Schultz N., Ritchie G.R., Eilbeck K. (2015). Improving the sequence ontology terminology for genomic variant annotation. J. Biomed. Semantics.

[22] Global Alliance for Genomics and Health (2016). A federated ecosystem for sharing genomic, clinical data. Science.

[23] Adams D., Altucci L., Antonarakis S.E., Ballesteros J., Beck S., Bird A., Bock C., Boehm B., Campo E., Caricasole A. (2012). BLUEPRINT to decode the epigenetic signature written in blood. Nat. Biotechnol..

[24] Bernstein B.E., Stamatoyannopoulos J.A., Costello J.F., Ren B., Milosavljevic A., Meissner A., Kellis M., Marra M.A., Beaudet A.L., Ecker J.R. (2010). The NIH roadmap epigenomics mapping consortium. Nat. Biotechnol..

[25] Zerbino D.R., Wilder S.P., Johnson N., Juettemann T., Flicek P.R. (2015). The ensembl regulatory build. Genome Biol..

[26] Harrow J., Frankish A., Gonzalez J.M., Tapanari E., Diekhans M., Kokocinski F., Aken B.L., Barrell D., Zadissa A., Searle S. (2012). GENCODE: the reference human genome annotation for The ENCODE Project. Genome Res..

[27] McCarthy D.J., Humburg P., Kanapin A., Rivas M.A., Gaulton K., Cazier J.-B., Donnelly P. (2014). Choice of transcripts and software has a large effect on variant annotation. Genome Med.

[28] Zhao S., Zhang B. (2015). A comprehensive evaluation of ensembl, RefSeq, and UCSC annotations in the context of RNA-seq read mapping and gene quantification. BMC Genomics.

[29] Frankish A., Uszczynska B., Ritchie G.R., Gonzalez J.M., Pervouchine D., Petryszak R., Mudge J.M., Fonseca N., Brazma A., Guigo R. (2015). Comparison of GENCODE and RefSeq gene annotation and the impact of reference geneset on variant effect prediction. BMC Genomics.

[30] Rodriguez J.M., Maietta P., Ezkurdia I., Pietrelli A., Wesselink J.-J., Lopez G., Valencia A., Tress M.L. (2013). APPRIS: annotation of principal and alternative splice isoforms. Nucleic Acids Res..

[31] Kumar P., Henikoff S., Ng P.C. (2009). Predicting the effects of coding non-synonymous variants on protein function using the SIFT algorithm. Nat. Protoc..

[32] Adzhubei I., Jordan D.M., Sunyaev S.R. (2013). Predicting functional effect of human missense mutations using PolyPhen-2. Curr. Protoc. Hum. Genet..

[33] Landrum M.J., Lee J.M., Riley G.R., Jang W., Rubinstein W.S., Church D.M., Maglott D.R. (2014). ClinVar: public archive of relationships among sequence variation and human phenotype. Nucleic Acids Res..

[34] Amberger J.S., Bocchini C.A., Schiettecatte F., Scott A.F., Hamosh A. (2015). OMIM.org: Online Mendelian Inheritance in Man (OMIM®), an online catalog of human genes and genetic disorders. Nucleic Acids Res..

[35] Welter D., MacArthur J., Morales J., Burdett T., Hall P., Junkins H., Klemm A., Flicek P., Manolio T., Hindorff L. (2014). The NHGRI GWAS Catalog, a curated resource of SNP-trait associations. Nucleic Acids Res..

[36] Rath A., Olry A., Dhombres F., Brandt M.M., Urbero B., Ayme S. (2012). Representation of rare diseases in health information systems: the Orphanet approach to serve a wide range of end users. Hum. Mutat..

[37] Malone J., Holloway E., Adamusiak T., Kapushesky M., Zheng J., Kolesnikov N., Zhukova A., Brazma A., Parkinson H. (2010). Modeling sample variables with an Experimental Factor Ontology. Bioinformatics.

[38] Köhler S., Doelken S.C., Mungall C.J., Bauer S., Firth H.V., Bailleul-Forestier I., Black G.C., Brown D.L., Brudno M., Campbell J. (2014). The Human Phenotype Ontology project: linking molecular biology and disease through phenotype data. Nucleic Acids Res..

[39] Schreiber F., Patricio M., Muffato M., Pignatelli M., Bateman A. (2014). TreeFam v9: a new website, more species and orthology-on-the-fly. Nucleic Acids Res..

[40] Veidenberg A., Medlar A., Löytynoja A. (2016). Wasabi: An integrated platform for evolutionary sequence analysis and data visualization. Mol. Biol. Evol..

[41] Keane T.M., Goodstadt L., Danecek P., White M.A., Wong K., Yalcin B., Heger A., Agam A., Slater G., Goodson M. (2011). Mouse genomic variation and its effect on phenotypes and gene regulation. Nature.

[42] Paten B., Herrero J., Beal K., Fitzgerald S., Birney E. (2008). Enredo and pecan: Genome-wide mammalian consistency-based multiple alignment with paralogs. Genome Res..

[43] Paten B., Herrero J., Fitzgerald S., Beal K., Flicek P., Holmes I., Birney E. (2008). Genome-wide nucleotide-level mammalian ancestor reconstruction. Genome Res..

[44] Dewey C.N. (2007). Aligning multiple whole genomes with mercator and MAVID. Methods Mol. Biol..

[45] Harrow J.L., Steward C.A., Frankish A., Gilbert J.G., Gonzalez J.M., Loveland J.E., Mudge J., Sheppard D., Thomas M., Trevanion S. (2014). The Vertebrate Genome Annotation browser 10 years on. Nucleic Acids Res..

[46] McLaren W., Gil L., Hunt S.E., Riat H.S., Ritchie G.R.S., Thormann A., Flicek P., Cunningham F. (2016). The ensembl variant effect predictor. Genome Biol..

[47] O'Leary N.A., Wright M.W., Brister J.R., Ciufo S., Haddad D., McVeigh R., Rajput B., Robbertse B., Smith-White B., Ako-Adjei D. (2016). Reference sequence (RefSeq) database at NCBI: current status, taxonomic expansion, and functional annotation. Nucleic Acids Res..

[48] Clarke L., Zheng-Bradley X., Smith R., Kulesha E., Xiao C., Toneva I., Vaughan B., Preuss D., Leinonen R., Shumway M. (2012). The 1000 Genomes Project: data management and community access. Nat. Methods.

[49] Barrett J.C., Fry B., Maller J., Daly M.J. (2005). Haploview: analysis and visualization of LD and haplotype maps. Bioinformatics.

[50] Hodgkins A., Farne A., Perera S., Grego T., Parry-Smith D.J., Skarnes W.C., Iyer V. (2015). WGE: a CRISPR database for genome engineering. Bioinformatics.

[51] Flicek P., Amode M.R., Barrell D., Beal K., Billis K., Brent S., Carvalho-Silva D., Clapham P., Coates G., Fitzgerald S. (2014). Ensembl 2014. Nucleic Acids Res..

[52] Raney B.J., Dreszer T.R., Barber G.P., Clawson H., Fujita P.A., Wang T., Nguyen N., Paten B., Zweig A.S., Karolchik D. (2014). Track data hubs enable visualization of user-defined genome-wide annotations on the UCSC genome browser. Bioinformatics.

